# Population pharmacokinetics of vancomycin in serum and cerebrospinal fluid: variability in cerebrospinal fluid concentrations despite targeted serum exposure

**DOI:** 10.1128/aac.00569-26

**Published:** 2026-06-15

**Authors:** Lucie Polášková, Tomáš Moravec, Veronika Krejčí, David Netuka, Ondřej Slanař, Martin Šíma

**Affiliations:** 1 Department of Clinical Pharmacy, Military University Hospital Prague 48235, Prague, Czech Republic; 2 Department of Neurosurgery and Neurooncology, First Faculty of Medicine, Charles University and Military University Hospital Prague 69729, Prague, Czech Republic; 3 Department of Pharmacology, First Faculty of Medicine, Charles University and General University Hospital in Prague 69729, Prague, Czech Republic; University of Houston, Houston, Texas, USA

**Keywords:** vancomycin, cerebrospinal fluid, glomerular filtration rate, Monte Carlo simulation, nonlinear mixed-effects modeling, therapeutic drug monitoring, CNS infection, neurosurgical patients

## Abstract

Vancomycin is a key component of empirical therapy for central nervous system (CNS) infections, yet dosing is guided by systemic pharmacokinetic/pharmacodynamic (PK/PD) targets that may not reflect drug exposure at the site of infection. Cerebrospinal fluid (CSF) penetration is variable, and the relationship between serum and CSF concentrations remains poorly defined. This study aimed to develop a population pharmacokinetic model simultaneously describing vancomycin concentrations in serum and CSF using real-world therapeutic drug monitoring (TDM) data and to characterize the relationship between systemic and CSF exposure. A retrospective observational study was conducted in adult neurosurgical patients (*n* = 64) with secondary meningitis. Vancomycin concentrations in serum (*n* = 133) and CSF (*n* = 100) were analyzed using nonlinear mixed-effects modeling. Monte Carlo simulations were performed to evaluate exposure across dosing regimens and renal function strata. Additional simulations under targeted serum exposure (AUC_24_ ~500 mg·h/L) with minimized interindividual variability in clearance were used to isolate variability in CSF exposure. A two-compartment model with a CSF compartment adequately described the data. Clearance was significantly associated with the estimated glomerular filtration rate. The median CSF-to-serum AUC ratio was 0.29, with substantial interindividual variability. Simulations demonstrated that, despite consistent serum exposure, CSF concentrations remained highly variable, with median (5^th^-95^th^ percentile) average CSF concentrations of 6.2 (3.2–10.4) mg/L. The probability of achieving higher CSF concentration thresholds declined markedly with increasing targets. Vancomycin CSF exposure exhibits substantial variability that is not reliably predicted by serum pharmacokinetics, even under standardized systemic exposure. These findings highlight limitations of serum-guided dosing for CNS infections and support the need for compartment-specific dosing strategies and PK/PD targets.

## INTRODUCTION

Central nervous system (CNS) infections, including bacterial meningitis and ventriculitis, remain associated with substantial morbidity and mortality despite advances in antimicrobial therapy. Empirical treatment regimens commonly include vancomycin to ensure coverage against resistant gram-positive pathogens, particularly *Streptococcus pneumoniae* and methicillin-resistant *Staphylococcus aureus* (1, 2). Current clinical practice is largely guided by systemic pharmacokinetic/pharmacodynamic (PK/PD) targets, with vancomycin area under the concentration–time curve to minimum-inhibitory concentration ratio (AUC/MIC) of 400–600 considered optimal for efficacy while minimizing toxicity (3, 4). However, these targets are derived from serum exposure and do not explicitly account for drug penetration into the CNS, the primary site of infection in meningitis.

Vancomycin penetration into cerebrospinal fluid (CSF) is known to be variable and influenced by multiple factors, including the integrity of the blood-brain barrier, degree of meningeal inflammation, and patient-specific physiological characteristics (5–7). Reported CSF-to-serum concentration ratios range widely, often between 0.1 and 0.5, but with substantial interindividual variability (6, 8). While inflammation may transiently increase permeability and enhance CSF penetration, this effect is heterogeneous and difficult to predict in individual patients (5, 7). Consequently, systemic exposure may not reliably reflect antimicrobial exposure at the site of infection.

Therapeutic drug monitoring (TDM) of vancomycin is routinely used to optimize systemic exposure, yet its utility for predicting CSF concentrations remains uncertain. Several studies have suggested a relationship between serum and CSF concentrations; however, this association is often weak and characterized by considerable unexplained variability (6, 9).

Importantly, much of the available evidence is derived from small cohorts, sparse sampling, or descriptive analyses that do not fully characterize the pharmacokinetic processes governing distribution into the CSF. Although a limited number of population pharmacokinetic studies with richer sampling have been reported in specific neurosurgical populations, these remain constrained by small sample sizes and specialized settings (10, 11). Population pharmacokinetic modeling provides a framework to quantify variability, identify covariates, and disentangle systemic and distributional determinants of CSF exposure, yet integrated models simultaneously describing serum and CSF concentrations remain scarce.

We, therefore, hypothesized that, even under conditions of comparable or targeted systemic exposure, vancomycin concentrations in CSF exhibit substantial variability driven predominantly by distributional processes, limiting the predictive value of serum pharmacokinetics as a surrogate for CSF exposure.

The primary objective of this study was to develop a population pharmacokinetic model of vancomycin simultaneously describing concentrations in serum and CSF using real-world TDM data from patients with suspected or confirmed secondary post-neurosurgical meningitis. A secondary objective was to use model-based simulations under standardized systemic exposure conditions to characterize the relationship between serum and CSF exposure and to quantify the extent of variability in CSF drug concentrations. Ultimately, this work aims to inform future efforts toward compartment-specific dosing strategies for this type of infection.

## MATERIALS AND METHODS

### Study design and ethics

An open-label, retrospective observational study was conducted in adult patients (aged ≥18 years) treated with vancomycin for secondary meningitis following neurosurgical procedures. All patients were admitted to the Department of Neurosurgery and Neurooncology at the Military University Hospital Prague between January 2017 and January 2026.

Inclusion criteria required the availability of at least one measured serum and one CSF vancomycin concentration obtained during the initial phase of therapy (defined as the first 4 days of treatment).

Given the retrospective design of the study and the exclusive use of routinely collected clinical data, the requirement for study-specific informed consent was waived. Only patients who had provided general consent for hospitalization and explicitly agreed to the use of their anonymized data for research purposes were included in the analysis. CSF samples were collected exclusively as part of routine clinical care in patients with secondary post-neurosurgical meningitis. Sampling was performed for standard monitoring of the infection course and treatment response (e.g., via external ventricular or lumbar drainage systems or clinically indicated lumbar puncture). No additional CSF sampling procedures were performed for research purposes. Vancomycin concentrations were measured from the residual material obtained during routine diagnostic sampling.

### Data retrieval

Clinical records of all patients who met the inclusion criteria were reviewed to obtain demographic, laboratory, and clinical data. Body mass index (BMI) was calculated as body weight (kg) divided by the square of height (m). Du Bois body surface area (BSA), ideal body weight (IBW), adjusted body weight (ABW), and Boer lean body mass (LBM) were calculated using standard formulas (12). The creatinine-based glomerular filtration rate was estimated for each patient using the Chronic Kidney Disease Epidemiology Collaboration (CKD-EPI) equation (13), both as a normalized value (eGFR_norm_: mL/min/1.73 m^2^) and as an absolute value (eGFR: mL/min). CSF biomarkers were also recorded, including the coefficient of energy balance (KEB): a CSF metabolic index reflecting the balance between lactate and glucose, CSF albumin, CSF total protein, and polymorphonuclear leukocyte count in CSF. KEB was calculated using the standard formula: KEB = 38-18 × (CSF lactate [mmol/L] / CSF glucose [mmol/L]).

The vancomycin dosing regimen, including administration times and infusion rates, was recorded. Vancomycin concentrations were measured as a part of routine TDM at the Department of Clinical Biochemistry of the Military University Hospital in Prague, and sampling times were also documented. Vancomycin levels were assessed in both serum and CSF.

Vancomycin concentrations were determined using an immunoturbidimetric method based on the kinetic interaction of microparticles in solution (KIMS) on the Roche Cobas 8000 analyzer (Roche, Basel, Switzerland). The same analytical method was applied for both serum and CSF samples. The lower limits of detection and quantitation were 1.5 mg/L and 4.0 mg/L, respectively, with an analytical measuring range of 4.0–80.0 mg/L. Only concentrations obtained during the initial phase of vancomycin therapy (the first 4 days of treatment) were included in the pharmacokinetic analysis. Later concentration-time points were not included due to sparse sampling and TDM-guided dose adjustments, which may introduce feedback-induced bias by violating the assumption of independence between dosing decisions and observed concentrations in standard population pharmacokinetic models.

### Individual data processing and statistics

Descriptive parameters—mean, median, and interquartile range (IQR)—were calculated using GraphPad Prism 8.2.1 software (GraphPad Inc., La Jolla, USA).

### Population pharmacokinetic analysis

The nonlinear mixed-effects modeling software Monolix Suite version 2024R1 (Lixoft SAS, Antony, France), implementing the Stochastic Approximation Expectation-Maximization (SAEM) algorithm, was used to analyze vancomycin concentration–time data in both serum and CSF. One-, two-, and three-compartment structural models with first-order elimination were evaluated. In addition, different structural configurations were explored, including models with and without drug CL from the CSF compartment. Model parameters were assumed to follow log-normal distributions. Interindividual variability (IIV) was tested for each parameter, and constant, proportional, and combined residual error models were compared. Model selection was based on changes in the objective function value (OFV), Akaike information criterion, and Bayesian information criterion, as well as visual inspection of goodness-of-fit (GOF) plots, shrinkage diagnostics, and relative standard errors (R.S.E.) of parameter estimates. Due to identifiability issues between distribution parameters, the volume of distribution of the CSF compartment (Vd_CSF_) was fixed to 0.15 L in the final model.

In the second step, potential covariates were evaluated using the base model. Age, height, body weight, ideal body weight (IBW), adjusted body weight (ABW), lean body mass (LBM), body surface area (BSA), eGFR_norm_, eGFR, KEB, CSF albumin, CSF total protein, and polymorphonuclear leukocyte count in CSF were tested as continuous covariates, while sex was evaluated as a categorical covariate. Initial covariate screening was performed based on graphical exploration and univariate analysis using Pearson’s correlation. Covariates identified in this step were further assessed using a manual stepwise forward inclusion and backward elimination procedure. A decrease in OFV of >3.84 points (χ² test, α = 0.05, 1 degree of freedom) was required for inclusion, whereas covariates were retained during backward elimination if their removal resulted in an OFV increase >6.64 points (*P* < 0.01). Additional selection criteria included physiological plausibility, R.S.E. <50%, and the absence of systematic bias in GOF plots. The influence of covariates on model parameters was tested using both linear and power functions.

Model adequacy was evaluated using GOF plots, including observed versus individual and population predictions, as well as individual weighted residuals (IWRES) versus time and individual predictions. Individual fits were also inspected. All GOF diagnostics were performed separately for both serum and CSF data. Model performance was further assessed using a prediction-corrected visual predictive check (pcVPC), performed separately for serum and CSF concentrations, to account for variability in dosing inherent to real-world TDM data. The pcVPC was based on 500 simulated data sets and displayed the 10th, 50th, and 90th percentiles of simulated concentrations overlaid with the observed data.

Model robustness and parameter precision were further evaluated using a nonparametric bootstrap (*n* = 500 replicates). The median values and 2.5^th^–97.5^th^ percentile ranges obtained from the bootstrap analysis were compared with the parameter estimates of the final model.

Because Vd_CSF_ was fixed to a physiological value of 0.15 L, the robustness of this assumption was assessed through sensitivity analyses using alternative fixed Vd_CSF_ values of 0.10 L and 0.20 L.

η-shrinkage was evaluated as 1−Var (η_i_)/ω^2^; where Var(η_i_) is the variance of individual empirical Bayes estimates and ω^2^ is the estimated population variance of the random effect. Values near 0 indicated limited shrinkage, while small negative values were interpreted as negligible shrinkage due to numerical variability.

### Monte Carlo simulations

The final population pharmacokinetic model for vancomycin, including residual unexplained variability, was imported into Simulx software (version 2024R1; Lixoft SAS, Antony, France) to perform Monte Carlo simulations (250 simulated data sets corresponding to 16,000 virtual subjects in total, reflecting the original covariate distribution). Simulations were conducted to evaluate vancomycin exposure in serum and CSF across clinically relevant dosing regimens and renal function strata. Renal function was represented by fixed eGFR values of 60, 90, and 130 mL/min. For each eGFR category, three dosing regimens were simulated: 1.5 g every 12 h, 1 g every 8 h, and 0.75 g every 6 h. The simulated dosing regimens were selected to represent commonly used intermittent clinical dosing strategies with varying dosing frequency and dose fractionation, while keeping the total daily dose constant. All dosing scenarios were simulated at steady state to evaluate exposure differences between dosing regimens under stabilized pharmacokinetic conditions.

To explore clinical applicability, the relationship between eGFR and vancomycin CL, as described in the final population pharmacokinetic model, was converted into a dosing nomogram targeting a serum exposure of AUC_24_ = 500 mg·h/L, corresponding to the mid-point of the recommended therapeutic range (3).

For subsequent simulations, the calculated daily dose was administered in three divided doses (every 8 h), and simulations were performed under steady-state conditions, corresponding to day 7 of therapy. To ensure consistent attainment of the target serum exposure with minimal variability across simulated individuals, interindividual variability in CL was fixed to 0 (ωCL = 0). This approach represents an idealized scenario and was applied to isolate variability in CSF exposure attributable to distributional processes rather than differences in systemic pharmacokinetics. Using this AUC-guided dosing strategy, additional simulations were performed to evaluate CSF exposure. Given the limited evidence supporting PK/PD targets for vancomycin in CSF, CSF exposure was characterized using average CSF concentrations (C_avg,CSF_), independent of the dosing interval and consistent with the AUC-based framework. The proportion of simulated individuals achieving C_avg,CSF_ greater than or equal to predefined concentration thresholds (2, 4, 6, 8, and 10 mg/L) was calculated to describe the distribution of CSF exposure, in a manner analogous to the probability of target attainment.

## RESULTS

### Study population

Sixty-four patients were eligible for inclusion in pharmacokinetic analysis. Demographic and laboratory data of patients are summarized in Table 1. None of the patients required extracorporeal membrane oxygenation or hemodialysis, although these were not defined as exclusion criteria.

**TABLE 1:** Demographic and laboratory data of the patients^*a*^^,*b*^

Sex M/F	36 (56.2)/28 (44.8)
Age (years)	55 (45–67.5) [18–82]
Height (cm)	175 (167–180) [154–197]
Body weight (kg)	85 (70–95) [48–140]
Ideal body weight (kg)	66 (58–75) [47–87]
Adjusted body weight (kg)	75 (65–82) [48–100]
Lean body mass (kg)	57 (49–65) [35–80]
Body surface area (m^2^)	2.03 (1.85–2.18) [1.51–2.62]
Body mass index (kg/m^2^)	26.5 (24.1–31.2) [16.6–46.2]
Serum creatinine (µmol/L)	61 (50–74) [23–124]
eGFR_norm_ (mL/min/1.73 m^2^)	106.5 (90.5–119.0) [53.4–138]
eGFR (mL/min)	119.8 (101.0–136.0) [56.2–185.5]
Coefficient of energy balance	1.05 (−57.4 to 17.4) [−2,259.5 to 31.5]
CSF albumin (g/L)	0.8 (0.4–1.1) [0–16.6]
CSF total protein (g/L)	1.39 (0.77–1.99) [0.09–28.94]
Polymorphonuclear leukocytes in CSF (cells/μL)	122.5 (26.5–937.5) [0–22,199]

^*a*^ Data are expressed as median (IQR) [range] or n (%).

^*b*^ CSF, cerebrospinal fluid; eGFR_norm_, glomerular filtration rate estimated according to the CKD-EPI formula and normalized to the body surface area; eGFR, absolute value of the glomerular filtration rate estimated according to the CKD-EPI formula.

A loading dose of 1,500–4,000 mg was administered in 53 patients. During the course of therapy, four patients received continuous infusion for the entire treatment period, seven patients received both continuous and intermittent infusions at different times, and 53 patients received only intermittent infusion. Intermittent doses ranged from 500 to 3,000 mg, with dosing intervals of 6, 8, 12, or 24 h. The choice of the dosing regimen was entirely at the discretion of treating physicians and clinical pharmacists as part of routine patient care; the study protocol did not influence dosing decisions.

A total of 133 measured vancomycin serum concentrations (1–4 per patient, mean 2.1 per patient) were included in the analysis. In cerebrospinal fluid, 62 quantifiable concentrations and 38 observations below the limit of quantification were included (1–4 per patient, mean 1.6 per patient). Observations below the limit of quantification were treated as censored data using the likelihood-based method implemented in Monolix, conceptually corresponding to the M3 approach.

Vancomycin concentrations were obtained during routine TDM, with sampling time points determined by clinical dosing schedules. Most samples represented pre-dose (trough) concentrations, typically 6, 8, or 12 h after the previous dose depending on the dosing interval, while some were peak or mid-interval samples. The timing and distribution of serum and CSF sampling are illustrated in Fig. S1.

### Population pharmacokinetic analysis

A two-compartment model with first-order elimination and a proportional residual error model for both serum and CSF concentrations provided the best description of the vancomycin concentration-time data. The pharmacokinetic model was parameterized in terms of the volume of distribution of the central (serum) compartment (Vd), clearance from the central compartment (CL), volume of distribution of the CSF compartment (Vd_CSF_), which was fixed to 0.15 L, intercompartmental clearance (Q), and clearance from the CSF compartment (CL_CSF_). The Monolix project file corresponding to the structural population pharmacokinetic model is available in the Supplementary data.

During covariate model building, absolute eGFR was identified as the most influential covariate on vancomycin CL, described using a power function. Inclusion of eGFR resulted in a decrease in the OFV of 23.5 points and was supported by physiological plausibility as well as improved GOF plots. No other tested demographic or laboratory variables showed a statistically significant effect on the pharmacokinetic parameters. The final covariate relationships, population pharmacokinetic parameter estimates, and bootstrap results are summarized in Table 2.

**TABLE 2:** Estimates of the final vancomycin population pharmacokinetic model and the bootstrap results based on 500 simulations^*a*^

	Final model		Bootstrap analysis
Parameter	Estimate	R.S.E. (%)	Median (2.5^th^–97.5^th^ percentile)
*Fixed effects* *Vd = Vd_pop × exp(ηVd,i*) *CL = CL_pop × (eGFR/120) ^β_CL_eGFR^ × exp(ηCL,i*) *Q = Q_pop × exp(ηQ,i*) *CL_CSF_ = CL_CSF__pop × exp(ηCL_CSF_,i*) *Vd_CSF_ was fixed to 0.15 L*			
Vd_pop (L)	115.1	10.7	108.3 (84.9–133.8)
CL_pop (L/h)	6.50	4.61	6.49 (5.94–7.09)
β_CL_eGFR	1.20	17.5	1.13 (0.80–1.42)
Q_pop (L/h)	0.0079	13.1	0.0064 (0.0031–0.0150)
CL_CSF__pop (L/h)	0.019	13.8	0.015 (0.007–0.039)
Between-subject variability (%)			
ωVd	41.0	19.5	40.0 (21.0–58.0)
ωCL	30.0	11.4	29.0 (22.0–36.0)
ωQ	31.0	43.9	40.0 (16.0–60.0)
ωCL_CSF_	42.0	22.4	36.0 (17.0–61.0)
Error model parameters			
Proportional (serum)	0.15	10.7	0.14 (0.11–0.19)
Proportional (CSF)	0.16	18.4	0.15 (0.073–0.22)

^*a*^ Vd, volume of central (serum) compartment; CL, systemic clearance from central compartment; Vd_CSF_, volume of cerebrospinal fluid; CL_CSF_, clearance from cerebrospinal fluid; Q, intercompartmental clearance between serum and cerebrospinal fluid; pop, typical value of parameter; β_CL_eGFR, effect of eGFR (mL/min) on CL; eGFR, glomerular filtration rate estimated according to the CKD-EPI formula; CSF, cerebrospinal fluid; η_i_, random effect on parameter (subject-specific deviation from the typical value); R.S.E., relative standard error; ω, interindividual variability of the parameter*.*

GOF plots demonstrated good agreement between observed and model-predicted concentrations for both serum and CSF data (Fig. S2), with no major trends observed in the residuals (Fig. S3). The pcVPC (Fig. S4), performed separately for serum and CSF, showed that the 10th, 50th, and 90th percentiles of simulated concentrations adequately captured both the central tendency and variability of the observed data. As shown in Table 2, parameter estimates were obtained with acceptable precision, with R.S.E. <44% for all estimated parameters. Shrinkage was low for all parameters (Vd: 2.17%, CL: −1.48%, Q: −0.24%, CL_CSF_: −2.34%), indicating good reliability of individual parameter estimates. The bootstrap analysis (500 replicates) confirmed the stability of the final model, with median bootstrap estimates in close agreement with the final model estimates and narrow 2.5^th^–97.5^th^ percentile ranges. Sensitivity analyses demonstrated limited sensitivity of CSF-related parameter estimates to the assumed Vd_CSF_ value within a physiologically plausible range. Varying Vd_CSF_ by approximately ±33% around the reference value (0.15 L) resulted in comparatively smaller changes in CL_CSF_ (−26% to +26%) and Q (−23% to +21%). Although these parameters are structurally linked to Vd_CSF_, the less-than-proportional changes support the relative robustness of the model estimates and did not alter the overall interpretation of CSF vancomycin disposition.

### Monte Carlo simulations

Simulated concentration-time profiles demonstrated that vancomycin exposure in both serum and CSF was influenced by renal function and dosing regimen (Fig. S5 and S6). As expected, higher eGFR values were associated with lower serum and CSF concentrations across all dosing scenarios, while more frequent dosing resulted in reduced peak-to-trough fluctuations without substantially altering overall exposure. Despite these differences in absolute concentrations, the CSF penetration ratio, expressed as AUC_CSF_/AUC_serum_, remained consistent across all simulated scenarios, with a median value of 0.29 (Table 3). No clinically relevant differences in penetration were observed between dosing regimens or eGFR categories. However, substantial interindividual variability in penetration was noted, with wide distributions across all scenarios (Fig. S7; Table 3).

**TABLE 3:** Cerebrospinal fluid penetration ratio (AUC_CSF_/AUC_serum_) across dosing regimens and eGFR values^*a*^^,*b*^

	eGFR = 60 mL/min	eGFR = 90 mL/min	eGFR = 130 mL/min
1.5 g q12h	0.29 (0.15–0.48) [0.05–0.73]	0.29 (0.15–0.48) [0.04–0.73]	0.29 (0.15–0.49) [0.044–0.73]
1 g q8h	0.29 (0.15–0.47) [0.069–0.71]	0.29 (0.15–0.48) [0.058–0.75]	0.29 (0.15–0.48) [0.051–0.75]
0.75 g q6h	0.29 (0.15–0.47) [0.06–0.76]	0.29 (0.15–0.48) [0.048–0.69]	0.29 (0.15–0.48) [0.06–0.71]

^*a*^ Data are expressed as median (5^th^–95^th^ percentile) [min–max].

^*b*^ eGFR, estimated glomerular filtration rate; AUC_CSF_, area under the cerebrospinal fluid concentration-time curve; AUC_serum_, area under the serum concentration-time curve.

**Fig 1:**
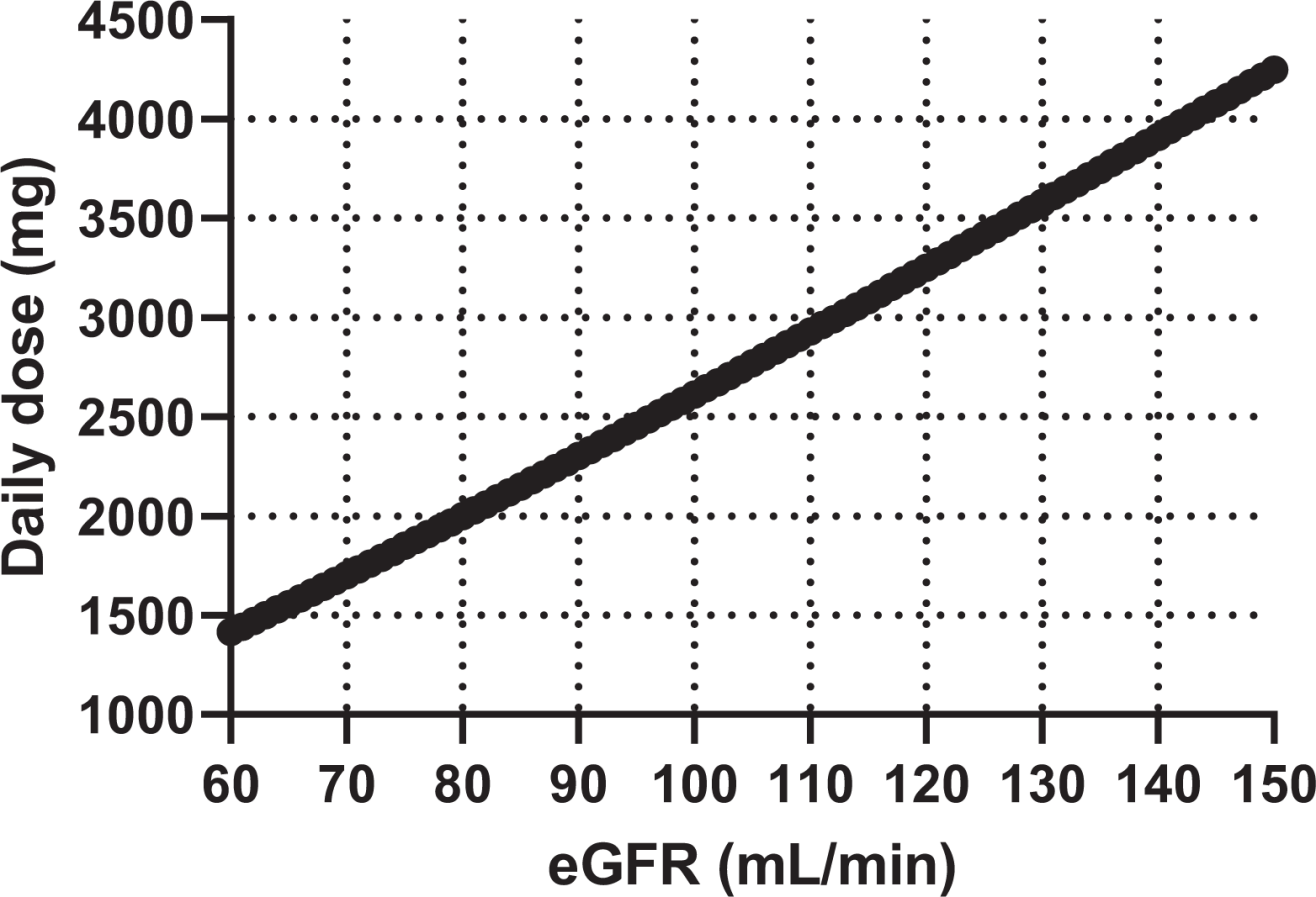
Model-derived nomogram for the selection of the optimal daily vancomycin dose based on the estimated glomerular filtration rate (eGFR). The optimal dose corresponds to a predicted serum exposure of AUC_24_ = 500 mg·h/L.

The nomogram developed based on the relationship between eGFR and vancomycin CL is presented in Fig. 1. It was used to determine the optimal daily dose according to renal function, defined as the dose achieving a target serum exposure of AUC_24_ = 500 mg·h/L.

**Fig 2:**
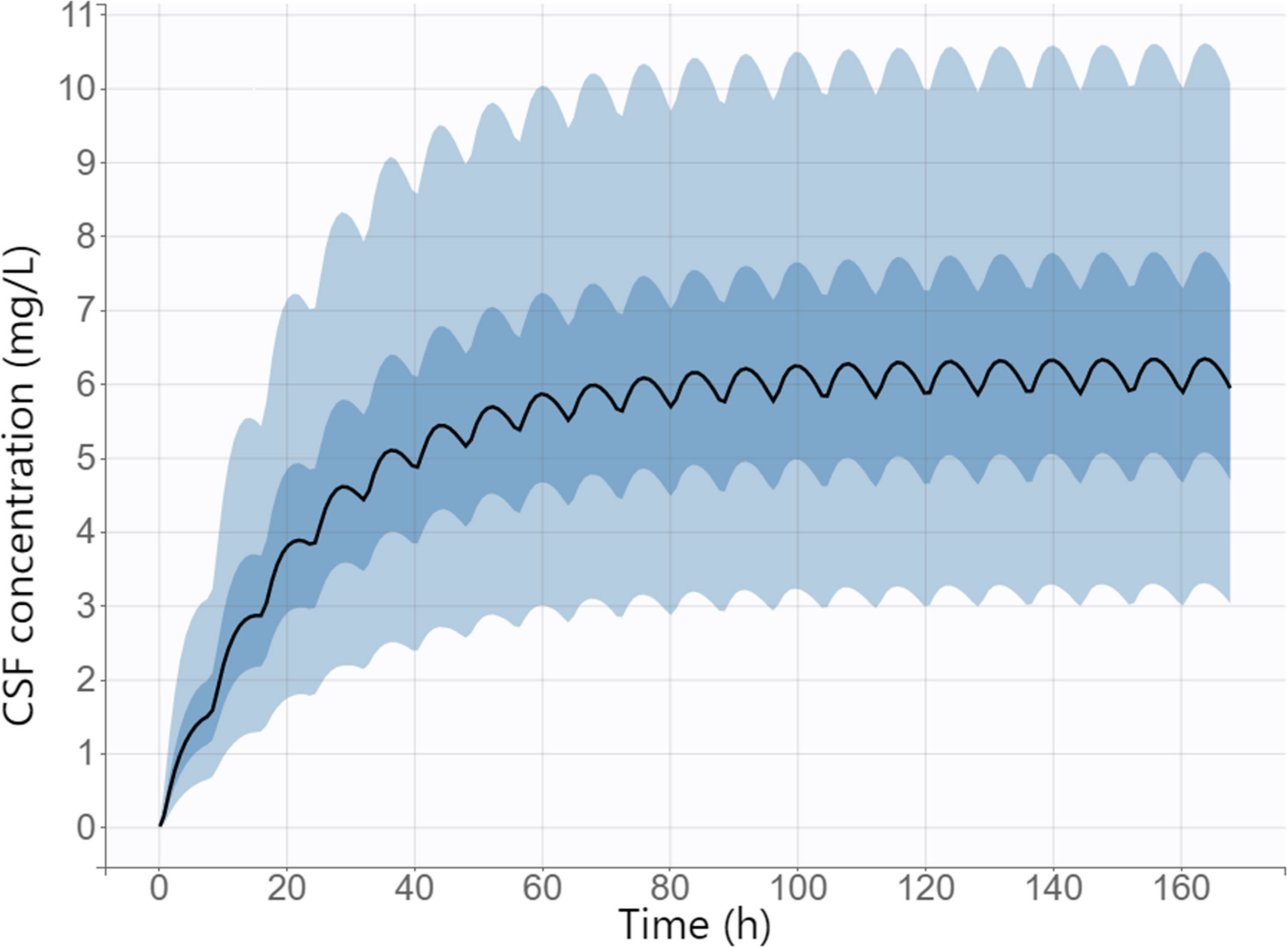
Simulated vancomycin cerebrospinal fluid (CSF) concentration-time profiles at steady-state serum exposure of 500 mg·h/L. The black line represents the median, and the four blue bands represent the percentiles (5%–27.5%, 27.5%–50%, 50%–72.5%, and 72.5%–95%) of the 90% simulated concentration distribution.

Using the AUC-guided dosing strategy targeting serum AUC_24_ = 500 mg·h/L with interindividual variability in CL fixed to 0 (ωCL = 0), simulated steady-state serum exposure showed minimal variability, with median (5^th^–95th^th^ percentile) AUC_24_ values of 499.4 (485.5–503.3) mg·h/L. In contrast, CSF exposure remained quite variable (Fig. 2). Median (5^th^–95^th^ percentile) C_avg,CSF_ values of 6.2 (3.2–10.4) mg/L were markedly lower than corresponding serum concentrations, 20.8 (20.2–21.0) mg/L, reflecting variability in CSF penetration.

The distribution of CSF exposure was further characterized by the proportion of individuals exceeding predefined concentration thresholds. The proportion achieving C_avg,CSF_ ≥2, 4, 6, 8, and 10 mg/L was 99.6%, 86.2%, 52.9%, 22.4%, and 6.7%, respectively, indicating a progressive decline in attainment with increasing thresholds.

## DISCUSSION

In this study, we developed a population pharmacokinetic model of vancomycin simultaneously describing serum and CSF concentrations using real-world TDM data from neurosurgical patients with CNS infections. The developed model, consisting of a central (serum) compartment and a CSF compartment, provided an adequate description of the observed data. Vancomycin CL was strongly associated with renal function, as described by eGFR, consistent with the well-established predominance of renal elimination (3, 14, 15). Renal function was parameterized using CKD-EPI eGFR, which is widely used in contemporary clinical practice and has been reported to provide reliable prediction of clearance for renally eliminated drugs, including vancomycin (14, 16). The estimated population CL (6.50 L/h at eGFR 120 mL/min) and its variability are in line with previously reported values in critically ill populations (17, 18). Similarly, the estimated population Vd (115 L) is consistent with expanded distribution volumes commonly observed in critically ill patients due to altered fluid balance and capillary leak (18, 19). An additional structural assumption of the model was the fixation of the Vd_CSF_ to 0.15 L. This value is consistent with the physiological volume of cerebrospinal fluid in adults, which is relatively well established and exhibits limited interindividual variability under most clinical conditions (5, 20). Fixing Vd_CSF_ was necessary to address identifiability issues between distribution parameters, a common challenge in models describing peripheral compartments with sparse data. This approach improved model stability and enabled more reliable estimation of Q and CL_CSF_. Importantly, the estimated CL_CSF_ (0.019 L/h) is in good agreement with physiological CSF turnover rates, which are typically reported to be approximately 0.3–0.4 mL/min (i.e., ~0.018–0.024 L/h) in adults (5, 21). This concordance supports the physiological plausibility of the model and suggests that CSF drug elimination is largely governed by bulk flow processes rather than active clearance mechanisms.

The estimated CSF penetration, expressed as the ratio of AUC_CSF_ to AUC_serum_, showed a median value of 0.29, which falls within the range reported in previous studies, typically between approximately 0.1 and 0.5 (5, 8, 9). However, consistent with prior observations, we identified substantial interindividual variability in CSF exposure. This variability persisted across dosing regimens and renal function strata, suggesting a predominant role of distributional processes rather than systemic pharmacokinetics.

Compared with previously published population pharmacokinetic studies in neurosurgical patients, our findings are broadly consistent but extend the existing knowledge in several ways. Prior models, often based on small cohorts (*n* ~ 15), have reported similar central pharmacokinetic parameters and variable CSF penetration (10, 11). However, these studies were typically limited to specific subpopulations, such as patients with external ventricular drains, and did not explore the relationship between systemic and CSF exposure under controlled conditions. By leveraging a larger real-world data set and performing simulation-based analyses, our study provides a more comprehensive characterization of CSF exposure variability.

The observed variability in CSF concentrations is physiologically plausible and reflects the complex and dynamic nature of drug distribution across the blood-brain and blood-CSF barriers. Vancomycin is a large, hydrophilic molecule with limited passive diffusion, and its penetration into the CNS is primarily governed by barrier permeability and bulk flow processes (5, 22). Inflammation of the meninges can increase permeability, leading to enhanced CSF penetration; however, this effect is heterogeneous and evolves over time (5, 7). As inflammation resolves, penetration may decrease, further contributing to temporal and interindividual variability. In addition, CSF pharmacokinetics are influenced by factors such as CSF turnover, the presence of external drainage systems, and local pathophysiological changes in neurosurgical patients. These factors are difficult to capture using standard clinical covariates, which may explain why no significant predictors of CSF distribution were identified in the present analysis. The relatively high interindividual variability observed for CSF-related parameters (Q and CL_CSF_) further supports the presence of substantial unexplained variability in CNS distribution processes. This is consistent with previous systematic evaluations reporting high variability in CSF penetration and the absence of reliable clinical predictors of CNS drug exposure (9).

The use of model-based simulations allowed separation of systemic and distributional sources of variability. Under an idealized scenario with interindividual variability in CL fixed to 0 (ωCL = 0) and dosing individualized using a nomogram to achieve a target serum exposure (AUC_24_ ~500 mg·h/L), CSF concentrations remained highly variable. This finding directly demonstrates that even optimal attainment of systemic PK/PD targets does not guarantee adequate CSF exposure.

From a clinical perspective, these results challenge the assumption that serum-based TDM is sufficient for optimizing therapy in CNS infections. While targeting AUC/MIC ratios of 400–600 is supported by evidence in bloodstream and other systemic infections (3), its applicability to CNS infections remains uncertain. Clinically, this implies that patients achieving guideline-recommended serum exposure may still experience suboptimal CSF concentrations, particularly when higher targets are required to overcome reduced CSF penetration or less susceptible pathogens.

The analysis of CSF exposure using C_avg,CSF_ further illustrates this point. While nearly all simulated patients achieved low concentration thresholds (≥2 mg/L), attainment decreased substantially for higher thresholds, with only a minority reaching concentrations ≥ 8–10 mg/L. This substantial interindividual variability in CSF exposure suggests that standard dosing may not achieve adequate CSF concentrations in all patients and supports the potential role of individualized dosing strategies and TDM.

The selected CSF concentration thresholds (2–10 mg/L) can be interpreted in the context of limited available data on vancomycin pharmacodynamics in the CNS. Effective bactericidal activity in CSF has been suggested to require concentrations several-fold above the MIC, with approximately 4× MIC often considered a relevant target (8). For typical vancomycin MIC values of 0.5–2 mg/L for relevant CNS pathogens, this corresponds to CSF concentrations of approximately 2–8 mg/L. Similarly, absolute CSF concentrations of 5–10 mg/L have been suggested in an earlier experimental study (23). Although these targets are not well validated and should be interpreted with caution, our simulation results indicate that attainment of higher CSF concentrations within this range is inconsistent, even when systemic exposure is optimized.

Interpretation of these findings is further complicated by the lack of well-defined PK/PD targets for vancomycin in the CSF. Unlike systemic infections, where AUC/MIC targets are supported by clinical and experimental data (3, 4), evidence for CNS infections remains sparse and largely indirect. Proposed approaches, such as maintaining CSF concentrations above the MIC throughout the dosing interval or extrapolating systemic AUC/MIC targets to the CSF compartment, have not been validated and do not account for the unique pharmacokinetic and pharmacodynamic conditions within the CNS (5, 6). In this context, our use of descriptive CSF exposure metrics represents a pragmatic strategy to characterize variability without relying on unvalidated targets. However, the identification of clinically relevant PK/PD indices in CSF remains a critical unmet need. Future studies integrating microbiological data, clinical outcomes, and CSF pharmacokinetics will be essential to define optimal therapeutic targets.

Several limitations should be acknowledged. First, the retrospective design and reliance on routine TDM data resulted in sparse and heterogeneous sampling. Although appropriate population pharmacokinetic methods were applied, this may still limit the precision of parameter estimates. Second, although the simulation approach allowed isolation of distributional variability, the assumption of zero interindividual variability in CL represents an idealized scenario that does not reflect clinical practice. Nevertheless, this approach was intentional and provided mechanistic insights into the determinants of CSF exposure. Third, the analyzed cohort consisted exclusively of patients with secondary post-neurosurgical meningitis; therefore, the findings should be interpreted primarily in the context of this type of CNS infection. No cases of encephalitis or isolated ventriculitis were present in the data set. In addition, CSF concentrations may not fully reflect vancomycin exposure in brain tissue or other intracranial compartments. Consequently, the findings should be interpreted as describing CSF penetration rather than overall CNS penetration. Fourth, CSF vancomycin concentrations were measured using a serum-validated immunoturbidimetric assay. While formal matrix-specific validation for CSF is limited, all measurements were performed in a certified clinical laboratory under routine conditions using the same analytical platform for serum and CSF samples. Fifth, the simulated renal function values (eGFR 60, 90, and 130 mL/min) were selected to represent clinically relevant states of impaired, normal, and augmented renal function within the observed range of the study population. Since the lowest observed eGFR in the cohort was 56.2 mL/min, simulations for substantially lower renal function values were not performed to avoid extrapolation beyond the available data. Consequently, the proposed dosing should not be directly extrapolated to patients with severe renal impairment. Finally, the lack of linked microbiological, clinical outcome, and toxicity data precluded the direct assessment of exposure-response and exposure-toxicity relationships in the CSF. In addition, the absence of an external validation cohort limits evaluation of the predictive performance of the model in independent patient populations. Therefore, while the model adequately describes CSF pharmacokinetic variability and supports simulation-based exploration of exposure scenarios, it should not be interpreted as a clinically validated dosing tool. Prospective studies incorporating microbiological, clinical outcomes, and toxicity data will be required to validate clinically relevant CSF PK/PD targets and evaluate the clinical applicability of the proposed simulation framework.

### Conclusion

To our knowledge, this study presents the largest population pharmacokinetic analysis of vancomycin simultaneously describing serum and CSF concentrations, based on real-world TDM data. Simulations were performed under an idealized scenario in which interindividual variability in systemic clearance was minimized, allowing isolation of variability in CSF exposure attributable to distributional processes. A key finding is the substantial heterogeneity in CSF concentrations despite consistent serum exposure, highlighting the limited predictability of CSF exposure from serum pharmacokinetics alone.

Using a descriptive approach based on average CSF concentrations, these results provide a quantitative characterization of CSF exposure and its variability and may support future efforts toward the development of compartment-specific dosing strategies and the identification of clinically relevant PK/PD targets in CSF—an area of substantial unmet need in the management of CNS infections.

## Data Availability

The data that support the findings of this study are available from the corresponding author upon reasonable request.

## SUPPLEMENTAL MATERIAL

Supplemental material aac.00569-26-s0001.pdf Fig. S1 to S7.

ASM does not own the copyrights to Supplemental Material that may be linked to, or accessed through, an article. The authors have granted ASM a non-exclusive, world-wide license to publish the Supplemental Material files. Please contact the corresponding author directly for reuse.
