# Supplementary material for: Population pharmacokinetics of vancomycin in serum and cerebrospinal fluid: variability in cerebrospinal fluid concentrations despite targeted serum exposure
Source: Antimicrob Agents Chemother. 2026 Jun 15;70(8):e00569-26. doi: 10.1128/aac.00569-26 (PMC13436342; doi:10.1128/aac.00569-26)

Supplementary data

Population pharmacokinetics of vancomycin in serum and cerebrospinal fluid: variability in cerebrospinal fluid concentrations despite targeted serum exposure

Monolix model file

```
[LONGITUDINAL]
input = {Vd, CL, Q, CL_CSF}

PK:
; fixed CSF volume
Vd_CSF = 0.15

; rate constants
k10 = CL / Vd
k12 = Q / Vd
k21 = Q / Vd_CSF
k20 = CL_CSF / Vd_CSF

; compartments
compartment(cmt=1, volume=Vd, concentration=Cs)
compartment(cmt=2, volume= Vd_CSF, concentration=Ccsf)

; administration
iv(cmt=1)

; elimination
elimination(cmt=1, k=k10)
elimination(cmt=2, k=k20)
```

```
; distribution
transfer(from=1, to=2, kt=k12)
transfer(from=2, to=1, kt=k21)

OUTPUT:
output = {Cs, Ccsf}
```

Supplementary figure S1. Observed vancomycin concentrations in (A) serum and (B) cerebrospinal fluid (CSF) showing the timing of sample collection during routine therapeutic drug monitoring (TDM).

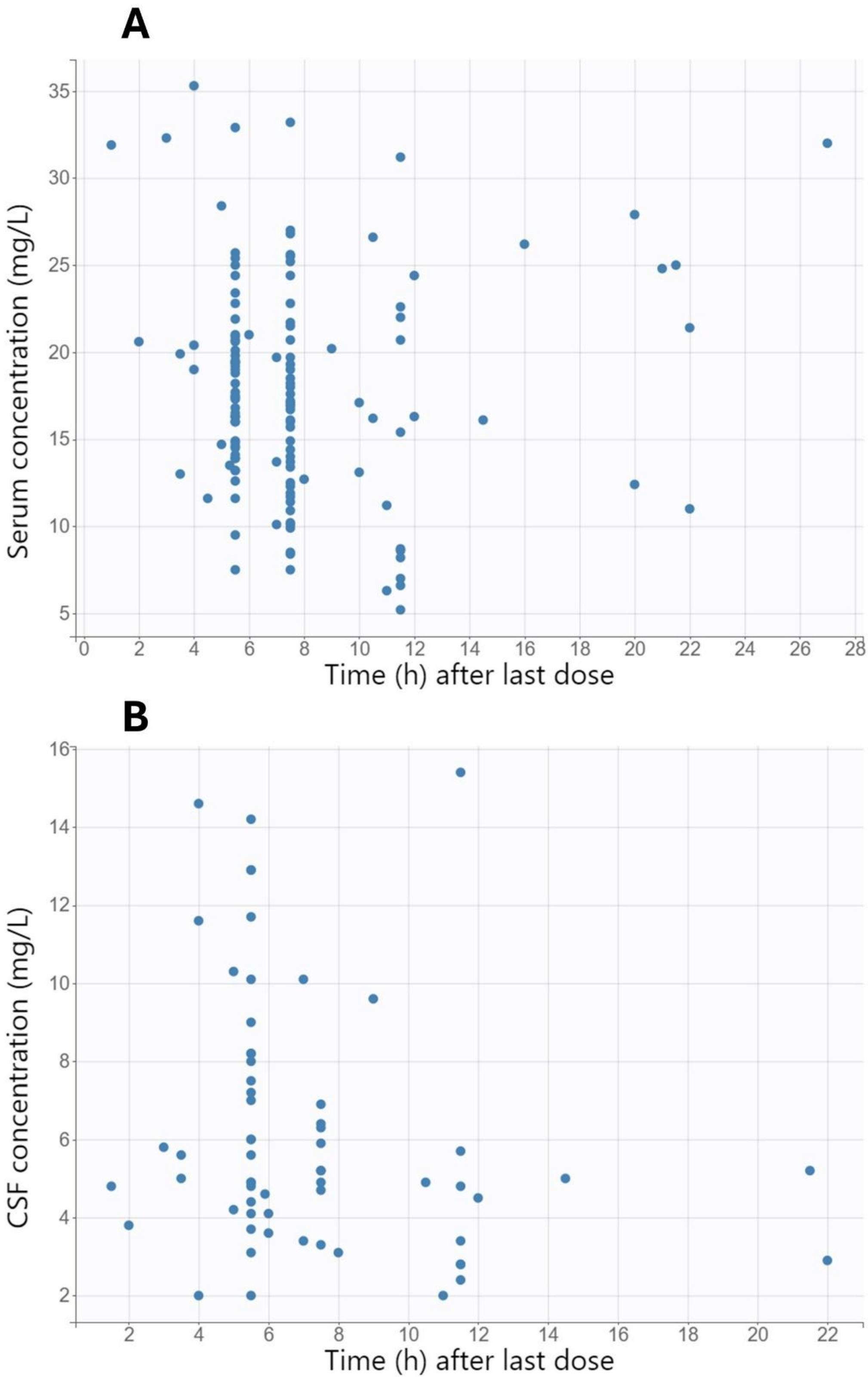

Supplementary figure S2. Goodness-of fit plots derived from the final vancomycin population pharmacokinetic model, illustrating population and individual predictions of vancomycin versus observed concentrations in (A) serum and (B) cerebrospinal fluid (CSF).

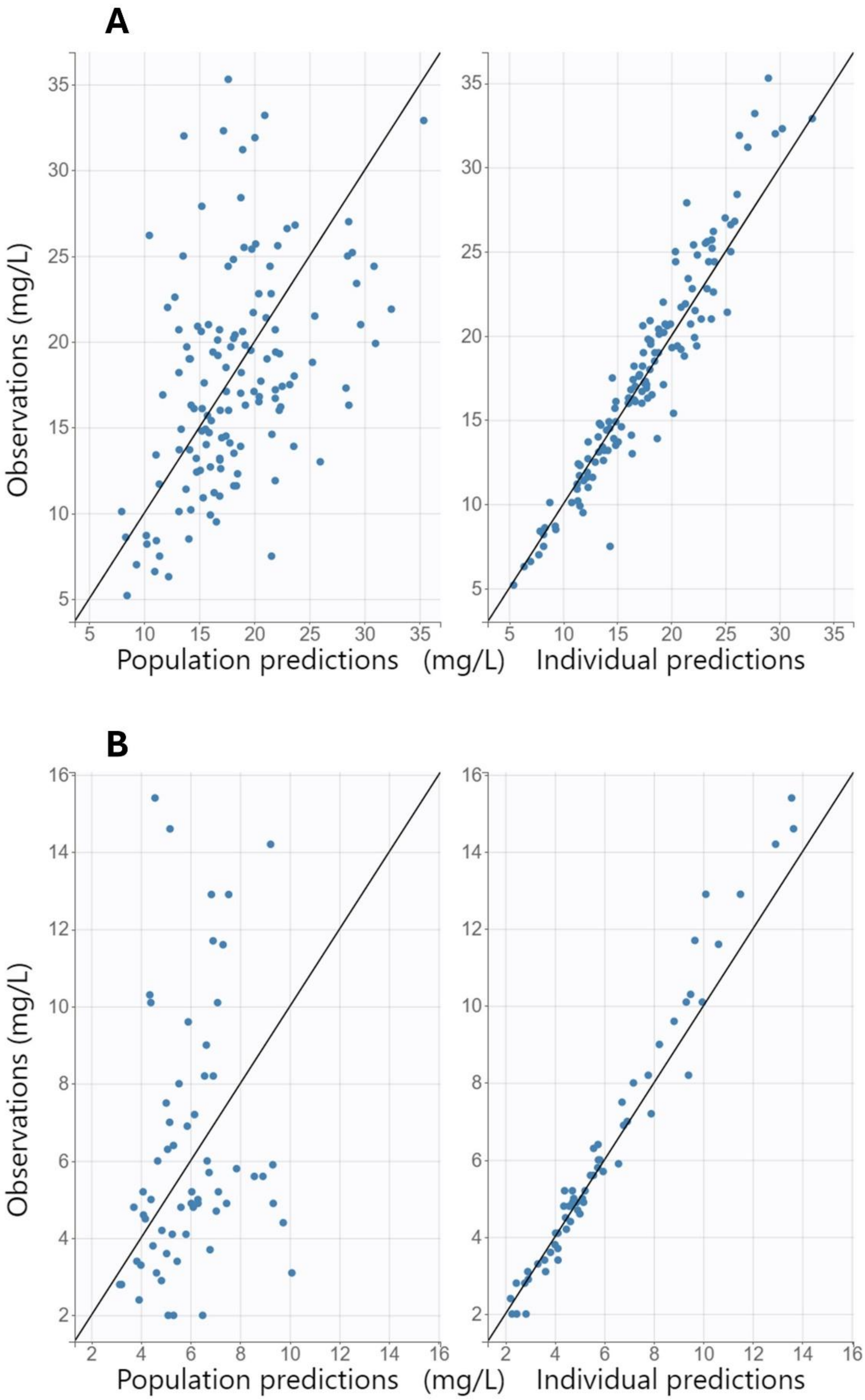

Supplementary figure S3. Individual weighted residuals (IWRES) from the final vancomycin population pharmacokinetic model. Panels show IWRES versus time and IWRES versus individual predictions in (A) serum and (B) cerebrospinal fluid (CSF).

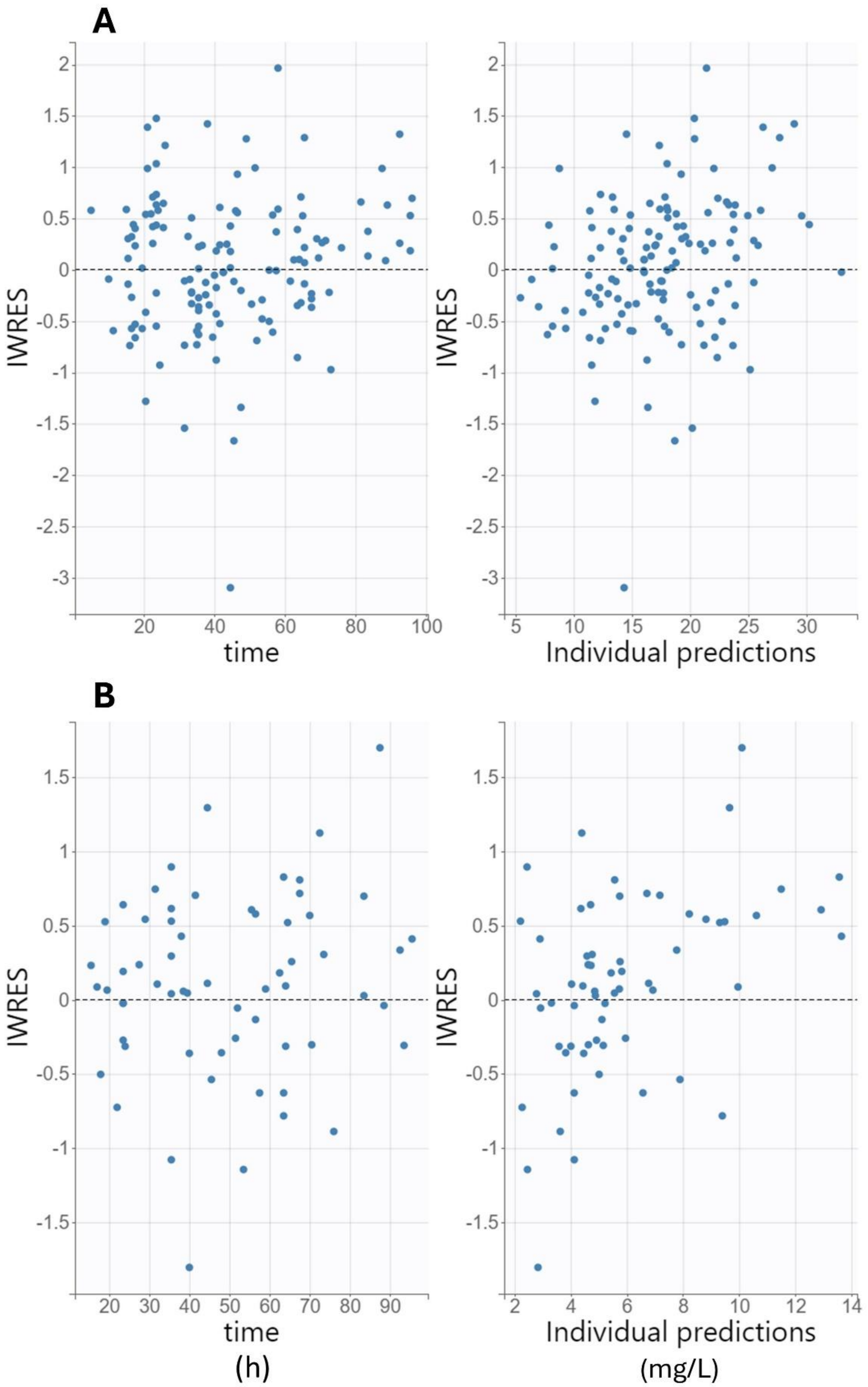

Supplementary figure S4. Prediction-corrected visual predictive check for vancomycin concentrations versus time from the final model. (A) serum; (B) cerebrospinal fluid (CSF). *Solid blue lines represent the 10th, 50th, and 90th percentiles of the observed data. Shaded areas represent the 90% confidence intervals of simulated data percentiles (10th – lower blue, 50th – pink, and 90th – upper blue.*

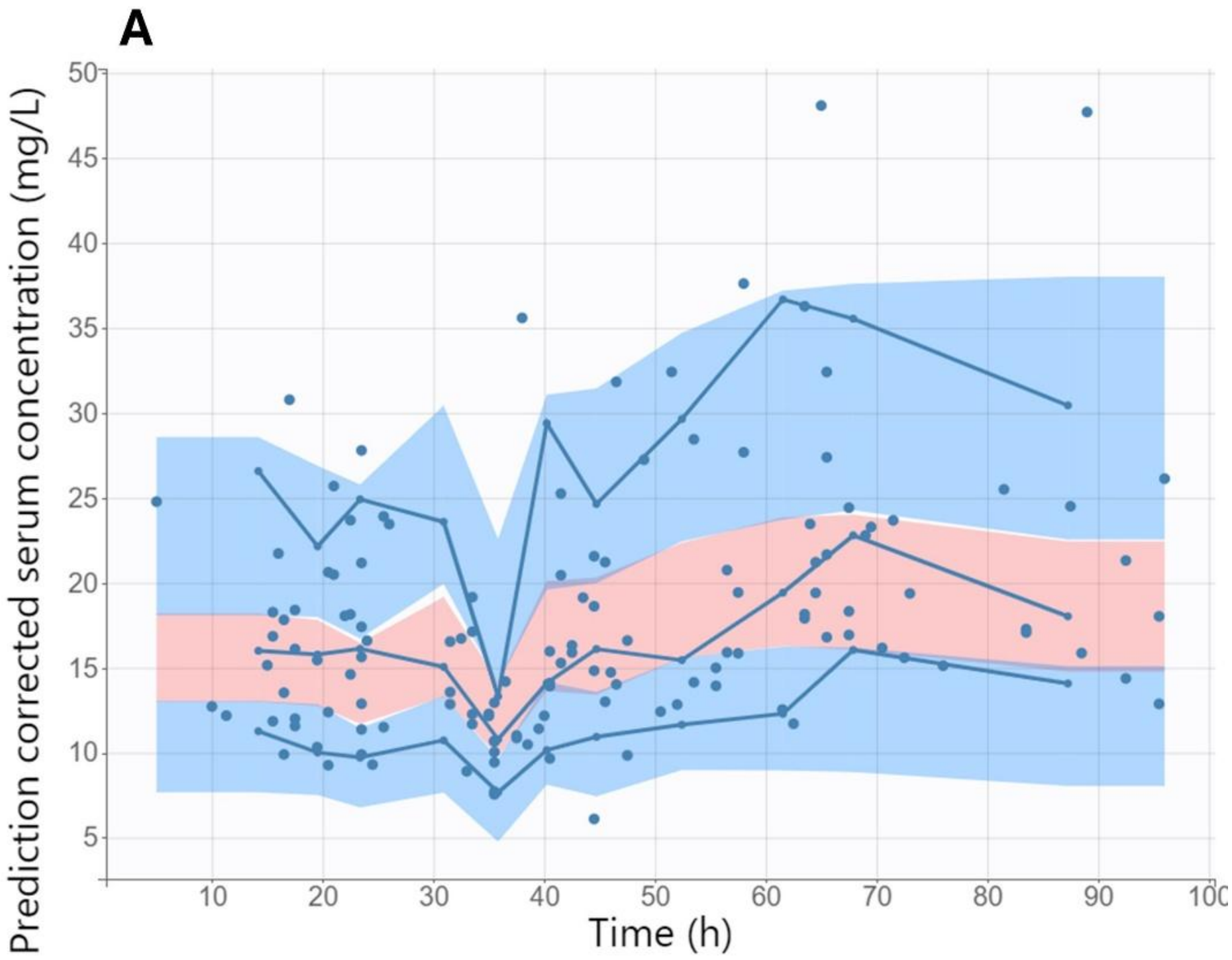

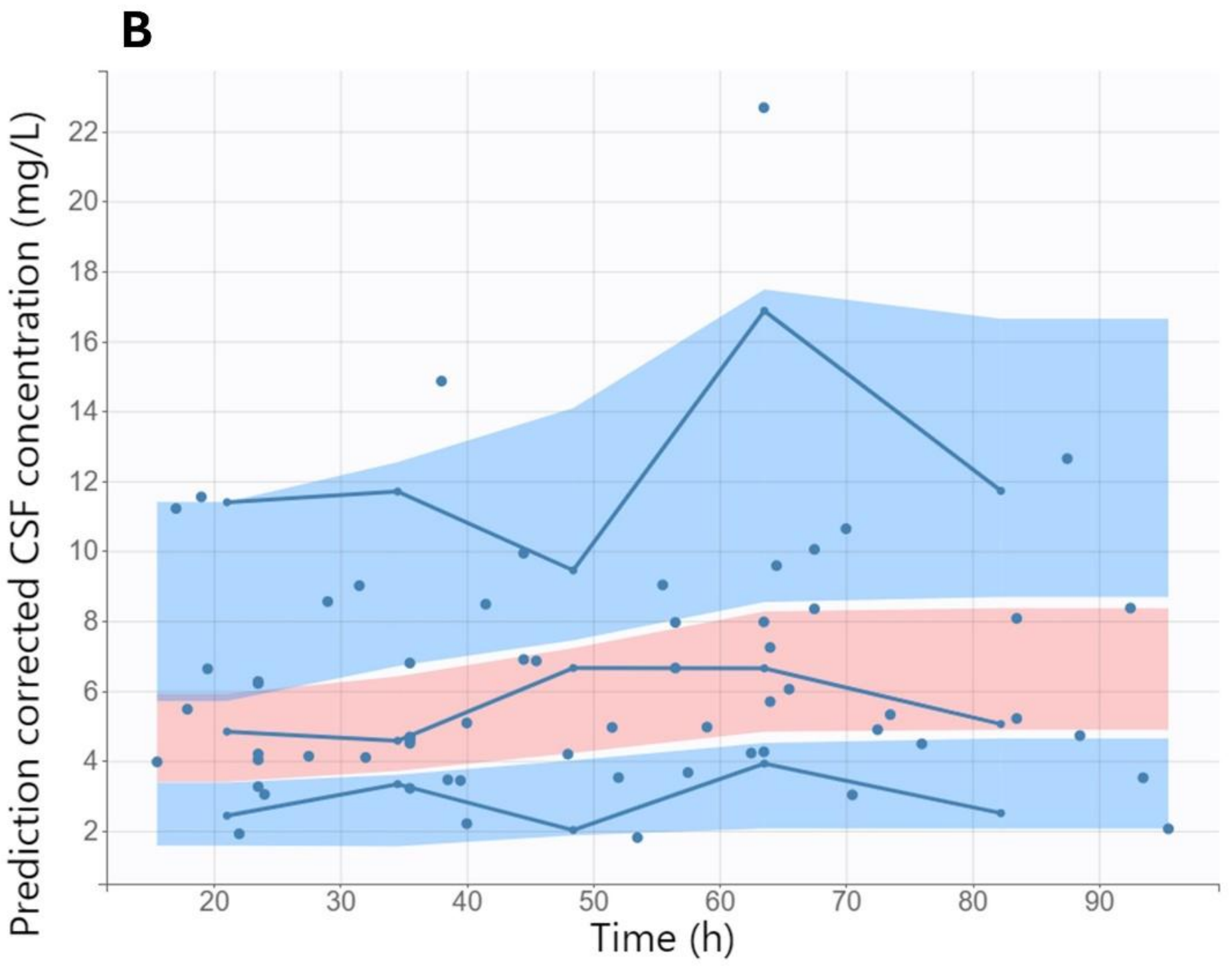

Supplementary figure S5. Simulated vancomycin serum concentration-time profiles for clinically relevant dosing regimens (1500 mg q12h, 1000 mg q8h, and 750 mg q6h) across renal function strata (eGFR of 60, 90, and 130 mL/min).

The black line represents the median, and the four blue bands represent the percentiles (5–27.5%, 27.5–50%, 50–72.5% and 72.5–95%) of the 90% simulated concentration distribution.

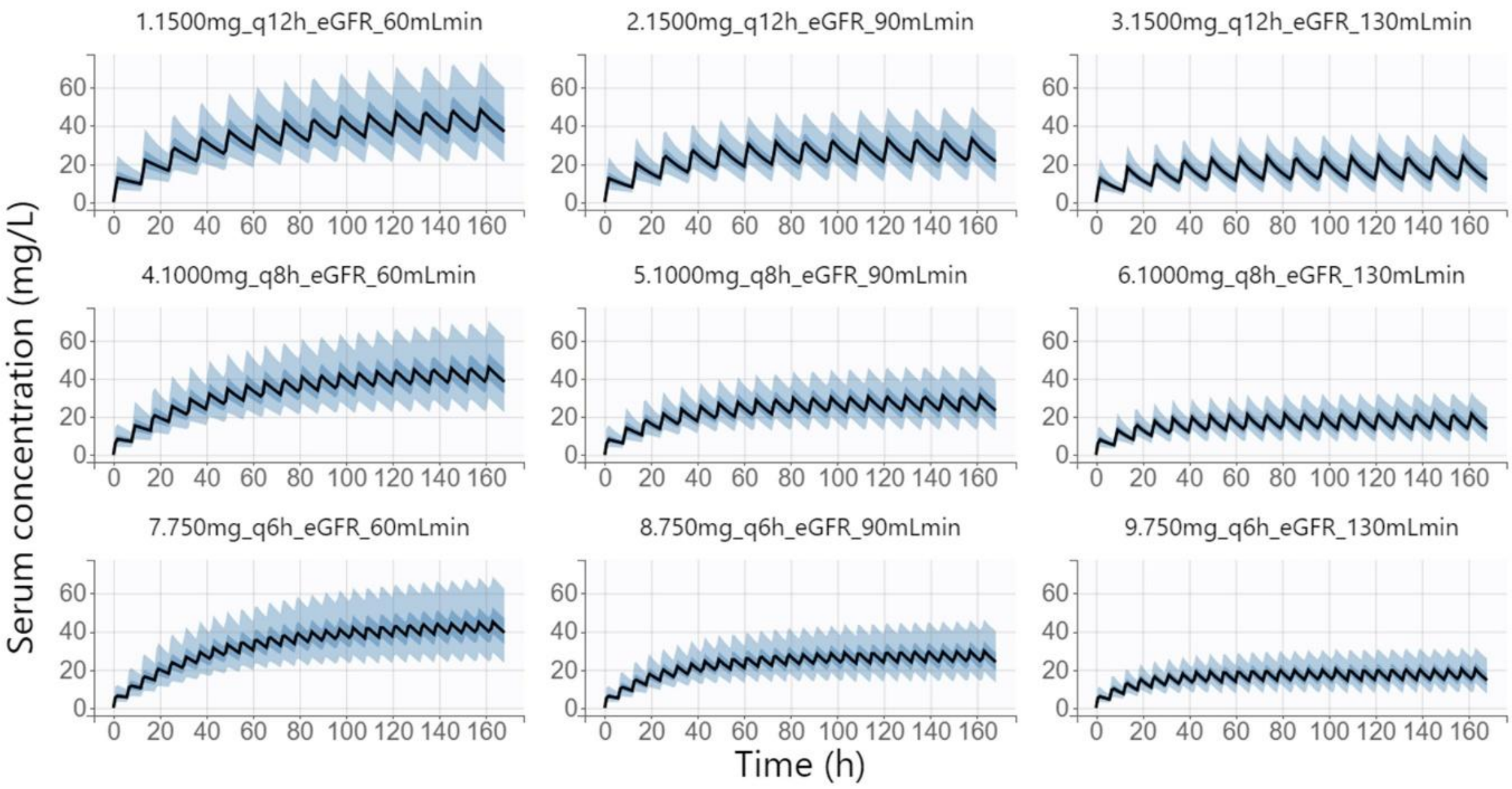

Supplementary figure S6. Simulated vancomycin cerebrospinal fluid (CSF) concentration-time profiles for clinically relevant dosing regimens (1500 mg q12h, 1000 mg q8h, and 750 mg q6h) across renal function strata (eGFR of 60, 90, and 130 mL/min).

The black line represents the median, and the four blue bands represent the percentiles (5–27.5%, 27.5–50%, 50–72.5% and 72.5–95%) of the 90% simulated concentration distribution.

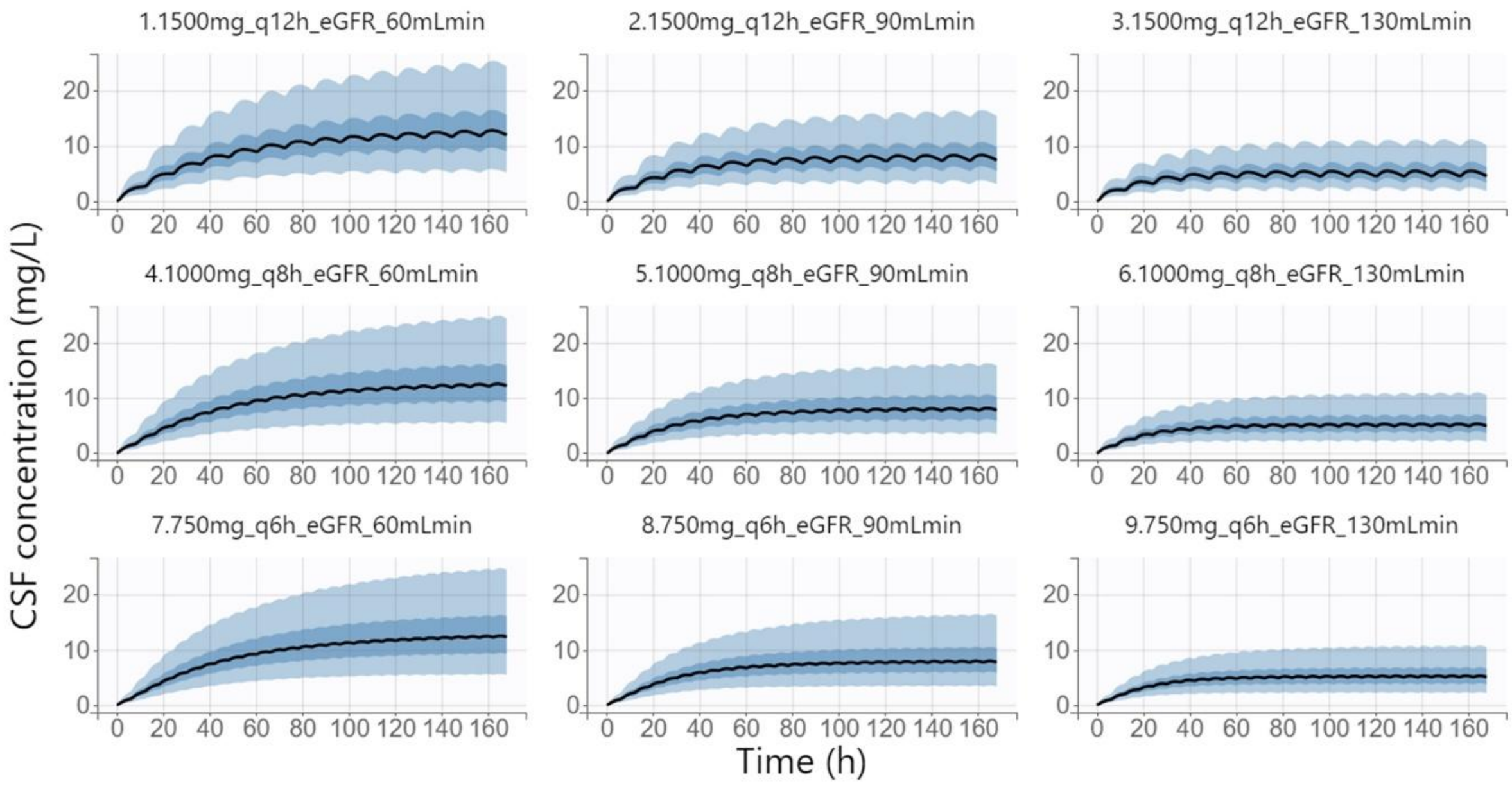

Supplementary figure S7. Simulated vancomycin cerebrospinal fluid (CSF) penetration ratio-time profiles (expressed as AUC_{CSF}/AUC_{serum}) for clinically relevant dosing regimens (1500 mg q12h, 1000 mg q8h, and 750 mg q6h) across renal function strata (eGFR of 60, 90, and 130 mL/min).

The black line represents the median, and the four blue bands represent the percentiles (5–27.5%, 27.5–50%, 50–72.5% and 72.5–95%) of the 90% simulated concentration distribution.

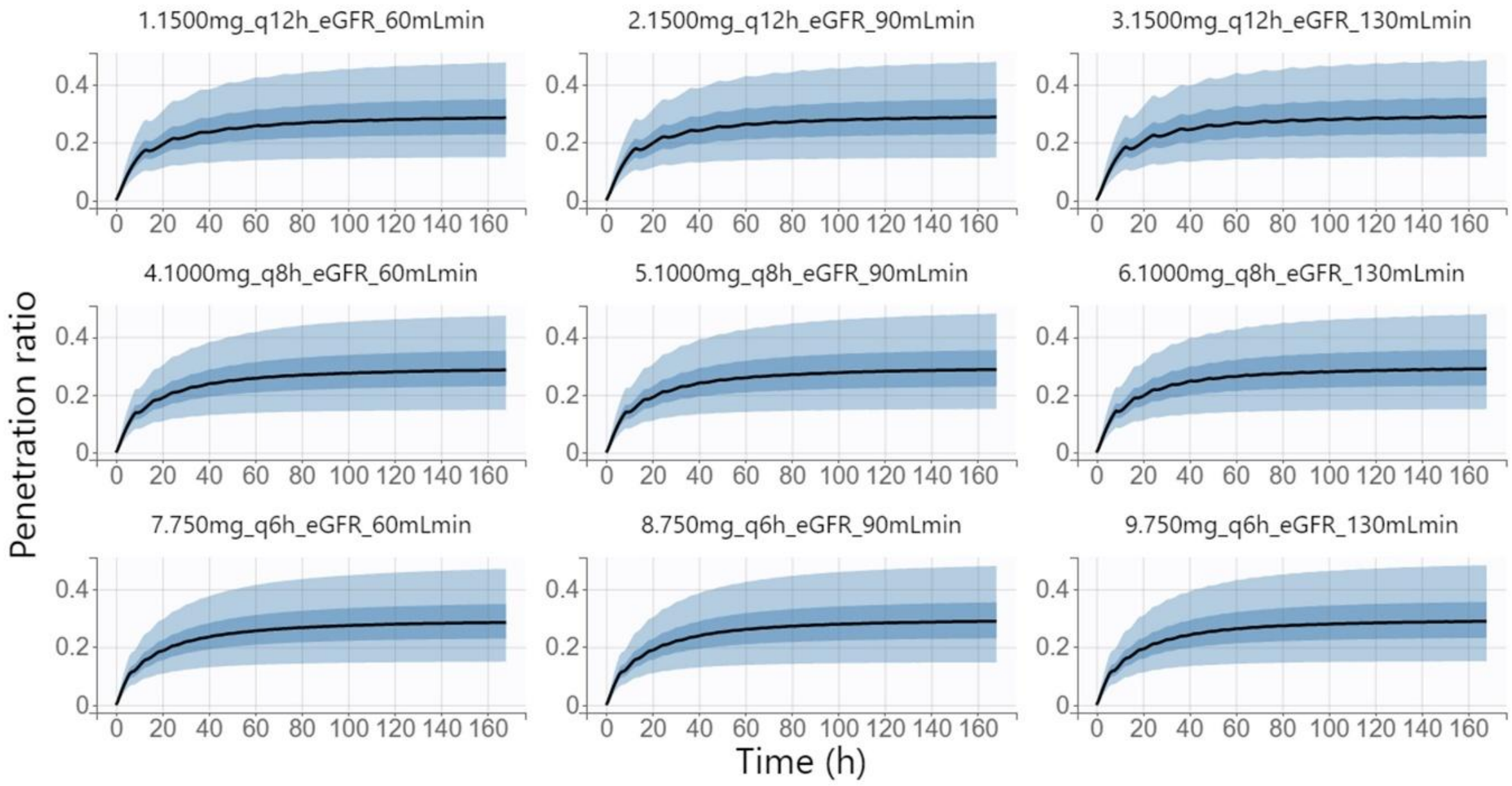

Supplement: Supplementary file 1 [file aac.00569-26-s0001.pdf]
